# XML-CIMT: Explainable Machine Learning (XML) Model for Predicting Chemical-Induced Mitochondrial Toxicity

**DOI:** 10.3390/ijms232415655

**Published:** 2022-12-09

**Authors:** Keerthana Jaganathan, Mobeen Ur Rehman, Hilal Tayara, Kil To Chong

**Affiliations:** 1 Department of Electronics and Information Engineering, Jeonbuk National University, Jeonju 54896, Republic of Korea; 2 School of International Engineering and Science, Jeonbuk National University, Jeonju 54896, Republic of Korea; 3 Advances Electronics and Information Research Center, Jeonbuk National University, Jeonju 54896, Republic of Korea

**Keywords:** mitochondrial toxicity, explainable machine learning, Mordred descriptors, predictive model, SHapley Additive exPlanations (SHAP)

## Abstract

Organ toxicity caused by chemicals is a serious problem in the creation and usage of chemicals such as medications, insecticides, chemical products, and cosmetics. In recent decades, the initiation and development of chemical-induced organ damage have been related to mitochondrial dysfunction, among several adverse effects. Recently, many drugs, for example, troglitazone, have been removed from the marketplace because of significant mitochondrial toxicity. As a result, it is an urgent requirement to develop in silico models that can reliably anticipate chemical-induced mitochondrial toxicity. In this paper, we have proposed an explainable machine-learning model to classify mitochondrially toxic and non-toxic compounds. After several experiments, the Mordred feature descriptor was shortlisted to be used after feature selection. The selected features used with the CatBoost learning algorithm achieved a prediction accuracy of 85% in 10-fold cross-validation and 87.1% in independent testing. The proposed model has illustrated improved prediction accuracy when compared with the existing state-of-the-art method available in the literature. The proposed tree-based ensemble model, along with the global model explanation, will aid pharmaceutical chemists in better understanding the prediction of mitochondrial toxicity.

## 1. Introduction

Cell toxicity caused by chemicals is a serious problem in the creation and use of chemicals such as medications, insecticides, chemical products, and cosmetics [1]. In recent decades, the initiation and development of chemical-induced organ damage have been related to mitochondrial dysfunction, among several adverse effects, as mitochondria are an important target for drug toxicity [2]. Mitochondria are widely termed to be the cell’s powerhouses. Mitochondria are found in almost all kinds of human cells and are critical to our life [3]. They are the biggest supplier of adenosine triphosphate (ATP) to human cells [4]. They are also engaged in other processes, such as cellular responses and cell death, commonly referred to as apoptosis [5]. Pathological reactions during tumour formation may alter mitochondrial mechanisms and activity.

In many situations of mitochondrial failure, oxidative phosphorylation is blocked, resulting in increased glycolysis and deadly lactate buildup in the blood [6]. Heart disease, neurological illness, kidney disease, and cancer have all been related to mitochondrial failure [7]. The liver, heart, and brain appear to be the primary victims of mitochondrial toxicity because they rely substantially on oxidative phosphorylation or act as the primary organs of drug metabolism. For instance, the anti-diabetic drug troglitazone was withdrawn because it was found to be hepatotoxic, causing mitochondrial damage and oxidative stress [8]. Several clinically used medicines have been linked to cardiotoxicity because they have direct effects on heart mitochondria [9]. Knowing how these chemicals interact with mitochondria can help to explain their toxicity or pharmacologic effects.

The functionality of mitochondria steadily deteriorates with ageing, and the prevalence of illnesses in the aged human body is significantly enhanced if the medicine includes chemicals that induce mitochondrial toxicity [10]. As a result, identifying and characterizing chemicals with mitochondrial toxicity is critical. Conventional in vivo and in vitro experiments are utilized to identify prospective mitochondrial toxins, but these procedures are time-consuming and costly [11,12].

In silico techniques, which use experimental data to develop a computational technique to screen out chemicals associated with mitochondrial toxicity, are faster and less costly than experimental procedures. However, because there has been little research on forecasting mitochondrial toxicity using computational approaches, it is critical to develop novel ways to anticipate mitochondrial toxicity [13,14].

In recent times, artificial intelligence has developed to a great extent, allowing it to solve multiple research problems [15,16]. However, for any artificial model, the dataset is of the utmost importance. Zhao et al. in [17] have gathered a vast number of chemicals linked with mitochondrial toxicity. They created 45 mitochondrial toxicity detection methods using five machine-learning algorithms and nine different kinds of molecular fingerprints. The reliability of prediction models were evaluated and verified using 10-fold cross-validation and an independent test set. Furthermore, the application scope of the estimation techniques was specified using the Euclidean distance approach. Finally, information gain and structural frequency analysis approaches were used to investigate structural alarms of compounds with mitochondrial toxicity.

Being inspired by the work of Zha et al. [17], we planned to improve the prediction model. In this work, we have proposed an explainable machine-learning model for predicting chemical-induced mitochondrial toxicity. The proposed model uses an optimized feature vector extracted from the Mordred feature descriptor [18]. Multiple classifiers were taken into account, including extreme gradient boosting (XGBoost) [19], light gradient-boosting machine (LightGBM) [20], CatBoost [21] and random forest (RF) [22]. Among all of them, CatBoost outperformed the others by a significant margin. Finally, the tree SHAP method [23] was applied to explain the proposed black box model predictions at the global level in order to rank the importance of key input Mordred descriptors that influence mitochondrial toxicity prediction results.

## 2. Dataset

The dataset utilized in this study is similar to the dataset used in [17]. The compounds linked to mitochondrial cytotoxicity were gathered from the PubChem database (AID 720635, 1347389, and 720637), DrugBank [24], and the literature [13]. For the negative dataset, drugs from DrugBank were selected that had been sold but had no linkage with mitochondrial toxicity or adverse effects. For positive data, the PubChem database [25] was used, where chemicals that produced a decrease in membrane potential are collected. In this work, the Simplified Molecular Input Line Entry System (SMILES) string notations are used to represent the molecular structure of all chemical compounds [26]. After canonicalizing the SMILES strings, the inorganic substances, metal-organic substances, mixes, salts, and duplicating compounds were deleted. The remaining dataset was then randomly separated into an 80% training dataset and a 20% testing dataset. Table 1 shows the summary of the datasets.

## 3. Methodology

**Figure 1:**
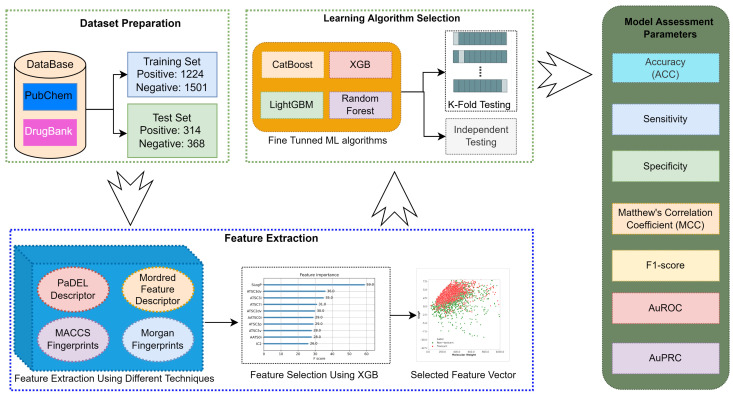
Development process of XML-CIMT model.

Figure 1 illustrates the development process of the XML-CIMT model. After preparing the training and testing datasets, the remaining workflow had 3 major steps, which are feature extraction, learning algorithm selection and model assessment. The molecular feature extraction and selection of machine learning algorithm for the model development are discussed in this section, and the details about the model performance assessment metrics are given in the next section.

### 3.1. Feature Extraction

In feature extraction, the first step was to decide which descriptor should be used to obtain the characteristics of the SMILES strings dataset. Molecular descriptors are commonly used to quantify the molecular properties of medicinal molecules. Using multiple free source programs, we can generate a large number of descriptors from the SMILES string format. In our case, we analyzed four different molecular descriptors, namely the PaDEL, MACCS, Morgan and Mordred feature descriptors, which were extracted by their freely available software [18,27,28].

Due to the small size of the dataset, we used these descriptors individually and tried to obtain the most optimized feature set. These descriptors mainly compute molecular markers and fingerprints. Next, 2D feature descriptors were extracted from open-source software. The Chemistry Development Kit was primarily used to compute these descriptors and fingerprints.

From the extracted feature descriptors, feature selection was carried out using the XGBoost feature selection technique. XGB is a gradient-boosting-based algorithm. One advantage of employing gradient boosting is that once the boosted trees are built, it is quite simple to obtain significance ratings for each feature. In general, significance offers a score indicating how useful or important each attribute was in the creation of the model’s bagged decision trees. The more frequently a property is utilized to make significant judgments with decision trees, the more important it is. This relevance was explicitly estimated for each property in the dataset, enabling attributes to be ordered and contrasted. We then encapsulated the model using the feature significance derived from the training data. This was used to pick attributes on the training data, train the model using the subset of attributes chosen, and then evaluate it on the test set employing a similar feature selection strategy.

**Figure 2:**
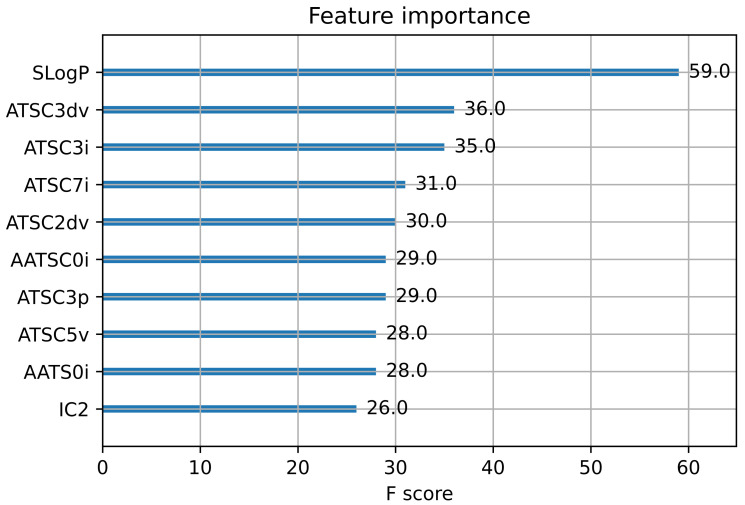
XGBoost-based feature importance graph from Mordred feature descriptors.

Figure 2 depicts a bar graph representing the feature importance scores of the top ten Mordred descriptors listed in descending order. The Wildman–Crippen LogP (SLogP) descriptor with the highest importance score is the most significant feature. The majority of the top ten 2D descriptors belong to the autocorrelation descriptor class. Table 2 lists the top ten descriptors’ names as well as their simplified descriptions. All of the Mordred descriptors are described in detail in the literature [18].

The selected features from the whole feature descriptor were then used to train the machine learning algorithm. Among all feature descriptors, the Mordred feature descriptor performed higher. Therefore, the best feature set of 267 features was determined after deleting features having less than 5% feature significant scores in the Mordred feature descriptor. The optimum set from the trained and independent sets was employed for model creation, internal validation, and external validation, in that order.

### 3.2. Learning Algorithms

Given a compound’s molecular descriptors as input, machine learning algorithms may predict mitochondrial toxicity. The tree-based ensemble methods outperformed other traditional machine learning classifiers (support-vector machine and logistic regression) among various techniques used to develop the prediction model for chemical mitochondrial toxicity [17]. Therefore, in this work, we have considered the following learning algorithms for our model performance comparison: extreme gradient boosting (XGBoost), light gradient-boosting machine (LightGBM), random forest (RF) and category boosting (CatBoost).

The group of boosting algorithms are collectively termed gradient boosting algorithms. The extreme gradient boosting package is frequently addressed as XGBoost. It is an open-source library for machine-learning tasks. It is an ensemble learning method that defines that in ensemble learning, multiple weak learners are combined to yield a strong learning method. XGBoost is a scale-able method with high speed and reliable output performance [29]. XGBoost can be deployed for both regression and classification purposes. XGBoost is capable of providing high speed as it is capable of carrying out parallel computations, which makes it 10 times faster than the traditional gradient boosting algorithm. XGBoost can take input data that contain unused elements in the set; such data are consequently termed sparse data. It takes sparse data for the tree booster and linear booster. XGBoost is particularly effective in the optimization of sparse data. Objective and evaluation functions can be customized, and such functions can be utilized in the XGBoost method. Another feature of XGBoost is that it is capable of providing regression, classification and ranking in machine learning [19]. Therefore, in this study, it is also used to obtain the feature importance values and to obtain the selected feature vector for the prediction.

LightGBM is an open-source library most commonly deployed for gradient boosting. LightGBM falls under the category of gradient boosting algorithms and is referred to as a light gradient boosting machine. LightGBM is a tree-based ensemble learning method that employs algorithms that are entirely based on histograms. This consequently increases the training speed, brings down the overall memory utilization, and ultimately provides parallel learning capability [20]. LigthtGBM can be deployed for both regression and classification problems. LightGBM employs a leaf-to-leaf approach in which a leaf with the maximum gain is identified. The main focus of LightGBM is to design such methods that are the least computationally intensive and are optimized in terms of computations. The instances with high gradients are sampled by employing a selective approach, which consequently provides higher performance while training the algorithm.

CatBoost, commonly termed categorical boosting, is an open-source gradient boosting library which primarily focuses on minimizing the prediction shift, which can take place in the course of the training process of an algorithm. CatBoost can be utilized for both regression and classification. CatBoost makes changes in the computations that are specifically performed on gradients [29]. This change consequently avoids the prediction shift during the training of the algorithm. This optimization improves the overall accuracy and efficiency of the model. CatBoost offers both types of implementations on GPUs and CPUs. The CatBoost implementation on the GPUs turns out to provide higher training and output performance when compared with the traditional gradient boosting method implementation on the GPUs [21].

Random forest is considered one of the most deployed and high-performing machine learning algorithms that provides both classification and regression [22]. Random forest is a supervised machine learning method based on an ensemble learning approach, meaning it comprises multiple weak learners who are grouped to form a strong learning method. The random forest comprises decision trees that are random and are generated by utilizing two differential sources of randomization. Random forest deploys multiple decision trees on different data samples; in the case of classification, majority voting is employed, and in regression, averaging is utilized. Random forest yields an optimum performance even when the hyper-parameters are set to default settings, which gives it the perception that the random forest approach is free of hyper-parameter constraints [30].

These machine-learning algorithms were fine-tuned. A grid search was employed to determine the best hyper-parameters for the model that will produce the most precise predictions. Grid searching is the technique of tweaking hyperparameters to find the ideal parameters for a particular model. The values of hyper-parameters have a substantial impact on prediction accuracy. Because there is no method to predict the optimum values for hyperparameters, we must preferably attempt all possible values to determine the optimal values. Because doing this manually might take a significant amount of time and resources, we utilize the grid search method to automate the adjustment of hyper-parameters.

## 4. Evaluation Metrics

The dependability and prediction capacity of the created models were evaluated in this study using 10-fold cross-validation and on the independent test dataset. Sensitivity, specificity, accuracy, Matthew’s correlation coefficient (MCC), and F1-score are the assessment indicators used in this study. The selected performance metrics are widely used in the literature for assessing classification models made for bioinformatics data [31]. These evaluation metrics are expressed mathematically in terms of the four outcomes of the error matrix: true positives (TP), true negatives (TN), false negatives (FN), and false positives (FP) [32]. Aside from these parameters, we evaluated the effectiveness of the XML-CIMT model using the area under the receiver-operating characteristic (AuROC) curve and the area under the precision-recall curve (AuPRC).

## 5. Results and Discussion

### 5.1. Data Analysis

**Figure 3:**
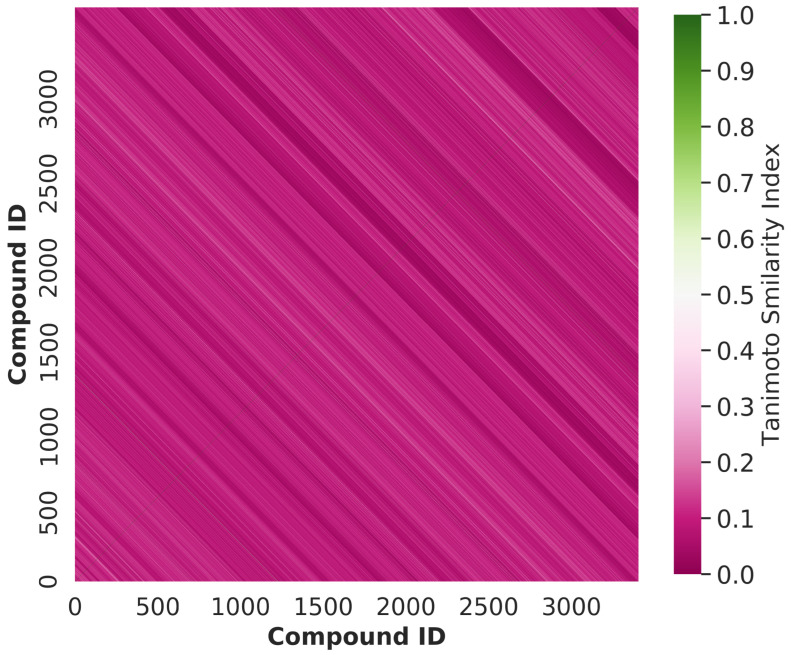
The heatmap represents the Tanimoto similarity index distribution of all compounds used in this study. The pink color represents the compounds with low similarity index, while the green color represents the compounds with high similarity index.

In this study, we analysed the structural diversity of all the chemical compounds by computing the the Tanimoto similarity index [33] based on the Morgan fingerprint with a radius of 2. In both the training and testing sets, a significant number of the compounds had resemblance indices that were lower than 0.30, with an average value of 0.0947. These findings imply that the chemical compounds in our dataset have greater structural diversity. Figure 3 shows a heatmap of the Tanimoto similarity index distribution of all compounds used in both the training and testing datasets. The heatmap is mostly pink, indicating that the molecules in the entire dataset have a significant amount of structural variation.

**Figure 4:**
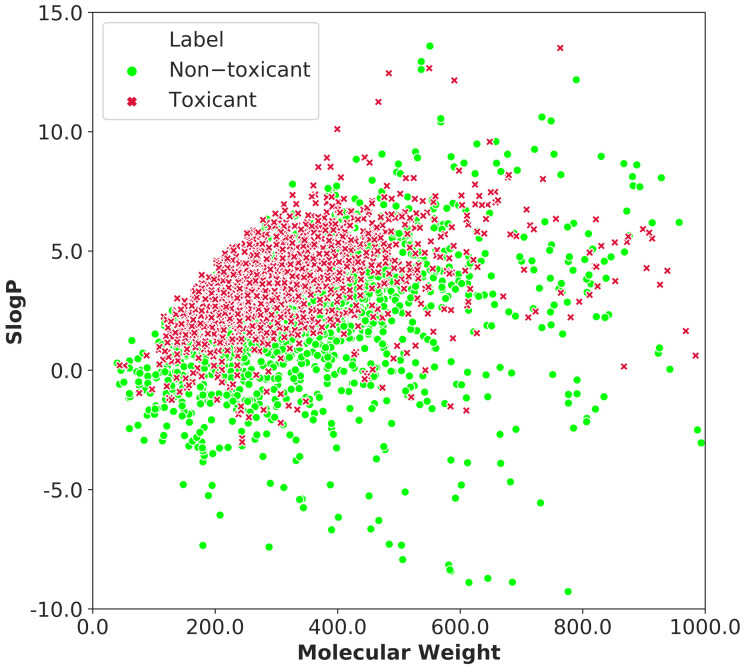
The chemical-space distribution of the whole dataset is defined by molecular weight and SLogP. The toxic substances are shown by red x markers, whereas the non-toxic compounds are represented by green circle markers.

We also investigated the chemical space distribution of the entire dataset using molecular weight and the octanol-water partition coefficient (SlogP), as shown in Figure 4. The molecular weight distribution ranges from 50 to 800, while the SLogP variation ranges from −5 to 8.5. It can be seen that the toxic and non-toxic compounds mostly share the same spatial distribution.

### 5.2. Comparison of Feature Descriptors

To select the best feature descriptor, we analyzed the performance of every descriptor after selecting the most important features among them. Table 3 shows the performance of different feature descriptors after feature selection. To achieve a fair comparison, a similar classifier was used, so for this case, we adopted the RF classifier. The results demonstrate that the classifier accuracy (84.26%), MCC (0.6826) and F1-score (81.5%) values of the Mordred feature descriptor are the best among all other descriptors used for performance comparison in this study. In the case of sensitivity, PaDEL showed a slightly better performance, but in parallel to that, the specificity had a huge drop, resulting in lower AuROC (area under the receiver-operating characteristic curve) and AuPRC (area under the precision-recall curve) values. Furthermore, we also combined all the descriptors and passed them by feature selectors and classifiers to see their performance. However, by combining all the descriptors, we were unable to improve the results when compared to the Mordred feature descriptor alone.

### 5.3. Comparison of Different Classifiers

Choosing the right ML algorithm for any task is an essential step towards performance. As discussed earlier. multiple classifiers are taken into account. Table 4 shows the comparison of the four best-performing classifiers on selected features from the Mordred feature descriptor. In the case of 10-fold cross-validation, CatBoost performed better in the case of accuracy, sensitivity, MCC and F1-score. However, in the case of specificity, AuROC, and AuPRC, the RF classifier outperformed the CatBoost classifier. It is important to notice here that the difference in the case of specificity, AuROC and AuPRC is very minute; however, for other metrics, CatBoost performed better with a greater margin.

Moving to the independent testing, it can be seen that CatBoost performed best for all the metrics. However, the RF classifier is even unable to achieve second-best results, as LightGBM performed much better than RF for the independent dataset, even though, in some cases, XGB showed improved results compared to RF. Keeping in view these analyses, we chose CatBoost as the classifier for XML-CIMT.

### 5.4. Comparison with Existing Technique

**Figure 5:**
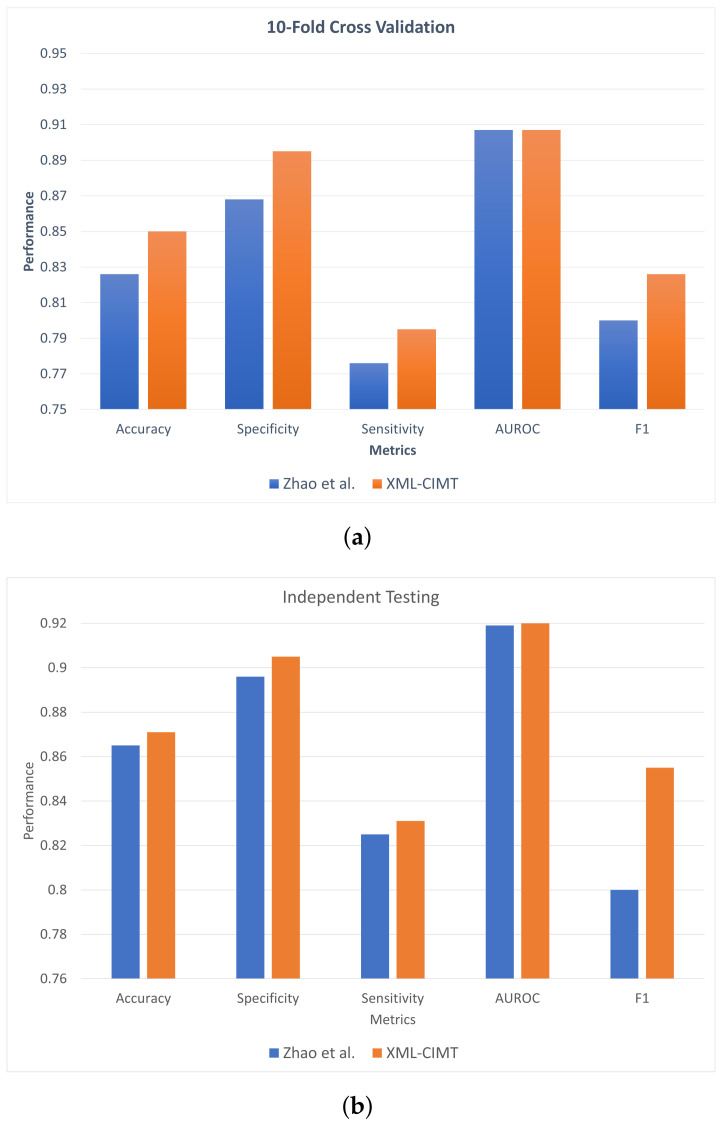
Performance comparison between XML-CIMT and Zhao et al. [17] model of results from (**a**) 10-fold cross validation and (**b**) independent testing.

The proposed model must perform better when compared with the existing state-of-the-art tool. Therefore, we compared our achieved results with the existing state-of-the-art results, which are by Zhao et al. [17]. Table 5 and Figure 5 illustrate the performance comparison between XML-CIMT and Zhao et al.’s model for 10-fold cross-validation as well as for independent testing.

As can be seen from Table 5 and Figure 5, the proposed XML-CIMT showcased improved performance for all the metrics. The independent dataset employed in this work, notably, did not contain any similar or highly comparable compounds present in the training dataset. Therefore, looking at the improvement in F1-score obtained by XML-CIMT for independent datasets illuminates that the proposed model is highly capable of learning the important features and discriminating them during the classification process.

### 5.5. Model Explanation and Descriptor Contribution

**Figure 6:**
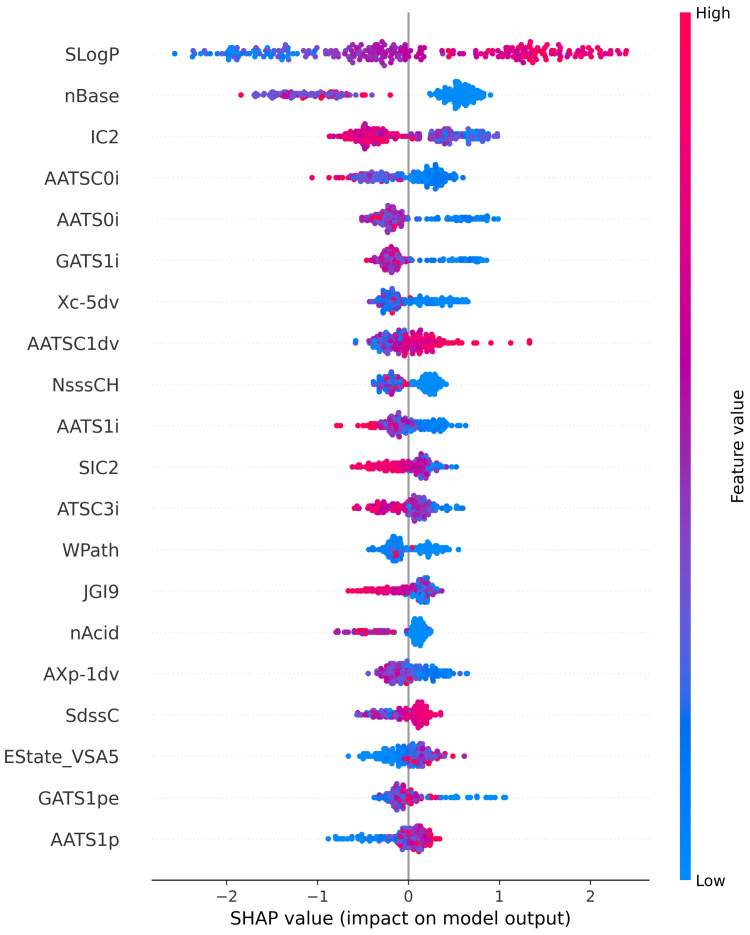
SHAP summary plot shows the contribution of the top 20 descriptors of the CatBoost model for mitochondrial toxicity prediction.

SHAP (SHapley Additive exPlanations) is a game-theoretic method for explaining the output of any supervised learning algorithm [34]. The Tree SHAP algorithm, in general, calculates precise SHAP values for machine learning models based on decision trees and their associated ensembles. In this study, the Tree SHAP explainer method is also used to further investigate the influence of the selected Mordred descriptors on the prediction of the proposed CatBoost model for chemical-induced mitochondrial toxicity. Figure 6 presents the top 20 key descriptors that influenced the predictions of the proposed model. Figure 2 shows only the feature importance scores, whereas the SHAP summary plot (Figure 6) combines the importance of the descriptors with their effects on predictions. Additionally, feature importance is always positive, whereas SHAP values can be both negative and positive. The SHAP summary plot displays the SHAP value on the *x*-axis and the most significant features of the model in descending order on the *y*-axis. High-value descriptors are represented in red, whereas low-value descriptors are highlighted in blue. A SHAP value of zero represents no descriptor contribution, whereas contributions increase as the SHAP value moves away from zero. Each circular dot in the plot represents a single chemical compound.

The SHAP summary plot demonstrates that Wildman–Crippen LogP (SLogp) is the most significant descriptor. Figure 6 shows that high values of the SLogP descriptor have a high positive contribution to the prediction of mitochondrial toxicity, while its low values have a significant negative impact. The second most important feature is basic group count descriptor (nBase). In the summary plot, the majority of the autocorrelation descriptors (AATSC0i, AATS0i, GATS1i, AATS1i and ATSC3i) have a negative correlation with the prediction of mitochondrial toxicity. The information content descriptors (IC2 and SIC2) also have a negative impact on the prediction of mitochondrial toxicity. All significant descriptor definitions can be found in the literature [18].

## 6. Conclusions

In this study, we proposed a prediction model for the in vitro results of mitochondrial toxicity using an explainable machine learning approach. The experiments suggested that the Mordred feature descriptor works better than other descriptors. Therefore, feature selection is applied to the Mordred feature descriptor to obtain the optimal feature subset with greater than 5% feature importance. The CatBoost ML model performed better than the other tree-based ensemble models used for comparison in this work. Additionally, the SHAP explanation technique was applied to provide more relevant explanations that strengthen the model’s prediction results and rank the important modelling descriptors that influence the prediction results. The methods described in the manuscript demonstrate how the ML technique may be used with in vitro experiments in early safety assessment throughout lead optimization. The integration of prediction findings and in vitro data acquired during the initial stages of drug discovery might boost confidence in general performance evaluation. The obtained experimental data may also be utilized to enhance the models’ training sets, allowing for the continual development of the biochemical applicability domain and, finally, the improved performance of the model. The codes and data are made available at the following link: https://github.com/Rehman1995/XML-CIMT.

## Figures and Tables

**Table 1:** Summary of the databases utilized in this study.

Dataset	Mitochondrial Toxicity	Number of Samples	Total Dataset Size
***Training***	Positive	1224	2725
	Negative	1501	
***Testing***	Positive	314	682
	Negative	368	

**Table 2:** List of top ten Mordred descriptors with their brief descriptions.

Descriptor Name	Description
SLogP	Wildman–Crippen LogP
ATSC3dv	Centered Moreau–Broto autocorrelation of lag 3 weighted by valence electrons
ATSC3i	Centered Moreau–Broto autocorrelation of lag 3 weighted by ionization potential
ATSC7i	Centered Moreau–Broto autocorrelation of lag 7 weighted by ionization potential
ATSC2dv	Centered Moreau–Broto autocorrelation of lag 2 weighted by valence electrons
AATSC0i	Averaged and centered Moreau–Broto autocorrelation of lag 0 weighted by ionization potential
ATSC3p	Centered Moreau–Broto autocorrelation of lag 3 weighted by polarizability
ATSC5v	Centered Moreau–Broto autocorrelation of lag 5 weighted by vdw volume
AATS0i	Averaged Moreau–Broto autocorrelation of lag 0 weighted by ionization potential
IC2	2-ordered neighborhood information content

**Table 3:** Comparison of Different Feature Descriptors. (Bold values represent high performance).

Descriptor	Accuracy	Specificity	Sensitivity	MCC	AuROC	AuPRC	F1
PaDEL	0.8161	0.8349	**0.7934**	0.6309	0.8857	0.8818	0.7949
MACCs	0.8378	0.8877	0.7763	0.6725	0.9016	0.9023	0.8107
Morgan	0.8275	0.896	0.7431	0.6518	0.8975	0.8941	0.7936
Mordred	**0.8426**	**0.8994**	0.7729	**0.6826**	**0.9109**	**0.9106**	**0.8150**

**Table 4:** Comparison of Different Classifiers on Selected Features from Mordred feature Descriptor. (Bold values represent high performance).

Testing	Classifier	Accuracy	Specificity	Sensitivity	MCC	AuROC	AuPRC	F1
*10-Fold* *Cross-Validation*	CatBoost	**0.8499**	0.8948	**0.7949**	**0.6971**	0.9069	0.9036	**0.8264**
	LightGBM	0.8444	0.8874	0.7916	0.6855	0.9068	0.9040	0.8204
	XGB	0.8418	0.8848	0.7892	0.6800	0.9045	0.9019	0.8174
	RF	0.8426	**0.8994**	0.7729	0.6826	**0.9109**	**0.9106**	0.8150
*Independent*	CatBoost	**0.8710**	**0.9048**	**0.8311**	**0.7415**	**0.9200**	**0.9238**	**0.8546**
	LightGBM	0.8651	0.8968	0.8279	0.7304	0.9129	0.9177	0.8496
	XGB	0.8563	0.8803	0.8276	0.7126	0.9134	0.9179	0.8402
	RF	0.8563	0.9023	0.8021	0.7136	0.9140	0.9126	0.8363

**Table 5:** Comparison of XML-CIMT with existing state-of-the-art model. (Bold values represent high performance).

Testing	Model	Accuracy	Specificity	Sensitivity	AuROC	F1
*10-Fold* *Cross-Validation*	Zhao et al. [17]	0.826	0.868	0.776	0.907	0.800
	XML-CIMT	**0.850**	**0.895**	**0.795**	**0.907**	**0.826**
*Independent*	Zhao et al. [17]	0.864	0.896	0.825	0.919	0.800
	XML-CIMT	**0.871**	**0.905**	**0.831**	**0.920**	**0.855**

## Sample Availability

Samples of the compounds are available from the authors.
